# Cucurbitacin E reduces IL-1β-induced inflammation and cartilage degeneration by inhibiting the PI3K/Akt pathway in osteoarthritic chondrocytes

**DOI:** 10.1186/s12967-023-04771-7

**Published:** 2023-12-04

**Authors:** Lin Wang, Hui Xu, Xin Li, Hongwei Chen, Haigang Zhang, Xunpeng Zhu, Zhijie Lin, Shilei Guo, Zhibo Bao, Haicheng Rui, Wei He, Hui Zhang

**Affiliations:** 1 Department of Orthopaedics, the First Affiliated Hospital of Anhui Medical University, Hefei, Anhui Province China; 2 School of Basic Medicine Sciences, Anhui Medical University, Hefei, Anhui Province China; 3 Anhui Medical University, Hefei, Anhui Province China

**Keywords:** Osteoarthritis, Cucurbitacin E, Cellular thermal shift assay, Molecular docking, Molecular dynamics simulation, PI3K/Akt, DMM

## Abstract

**Background:**

Osteoarthritis is a degenerative joint disease. Cartilage degeneration is the earliest and most important pathological change in osteoarthritis, and persistent inflammation is one of the driving factors of cartilage degeneration. Cucurbitacin E, an isolated compound in the Cucurbitacin family, has been shown to have anti-inflammatory effects, but its role and mechanism in osteoarthritic chondrocytes are unclear.

**Methods:**

For in vitro experiments, human chondrocytes were stimulated with IL-1β, and the expression of inflammatory genes was measured by Western blotting and qPCR. The expression of extracellular matrix proteins was evaluated by immunofluorescence staining, Western blotting and saffron staining. Differences in gene expression between cartilage from osteoarthritis patients and normal cartilage were analysed by bioinformatics methods, and the relationship between Cucurbitacin E and its target was analysed by a cellular thermal shift assay, molecular docking analysis and molecular dynamics simulation. For in vivo experiments, knee osteoarthritis was induced by DMM in C57BL/6 mouse knee joints, and the effect of Cucurbitacin E on knee joint degeneration was evaluated.

**Results:**

The in vitro experiments confirmed that Cucurbitacin E effectively inhibited the production of the inflammatory cytokine interleukin-1β(IL-1β) and cyclooxygenase-2 (COX-2) by IL-1β-stimulated chondrocytes and alleviates extracellular matrix degradation. The in vivo experiments demonstrated that Cucurbitacin E had a protective effect on the knee cartilage of C57BL/6 mice with medial meniscal instability in the osteoarthritis model. Mechanistically, bioinformatic analysis of the GSE114007 and GSE117999 datasets showed that the PI3K/AKT pathway was highly activated in osteoarthritis. Immunohistochemical analysis of PI3K/Akt signalling pathway proteins in pathological slices of human cartilage showed that the level of p-PI3K in patients with osteoarthritis was higher than that in the normal group. PI3K/Akt were upregulated in IL-1β-stimulated chondrocytes, and Cucurbitacin E intervention reversed this phenomenon. The cellular thermal shift assay, molecular docking analysis and molecular dynamics experiment showed that Cucurbitacin E had a strong binding affinity for the inhibitory target PI3K. SC79 activated Akt phosphorylation and reversed the effect of Cucurbitacin E on IL-1β-induced chondrocyte degeneration, demonstrating that Cucurbitacin E inhibits IL-1β-induced chondrocyte inflammation and degeneration by inhibiting the PI3K/AKT pathway.

**Conclusion:**

Cucurbitacin E inhibits the activation of the PI3K/AKT pathway, thereby alleviating the progression of OA. In summary, we believe that Cucurbitacin E is a potential drug for the treatment of OA.

**Supplementary Information:**

The online version contains supplementary material available at 10.1186/s12967-023-04771-7.

## Introduction

Osteoarthritis (OA), also known as degenerative osteoarthritis [1], is a common chronic joint disease characterized by a series of biochemical and morphological changes, including destruction of articular cartilage, formation of redundant bone, sclerosis of subchondral bone and inflammatory hypertrophy of the synovial membrane that often occurs in weight-bearing joints such as the hip, knee and interphalangeal joints. The development and progression of OA is associated with many risk factors, including obesity, inflammation, trauma, genetics and abnormal biomechanics, among others, which can cause abnormalities in the chronic microenvironment and in turn induce degeneration of the joint [2]. The prevalence of OA is increasing due to the ageing of the population and the increasing number of people with obesity; according to WHO statistics, approximately 10% of people over 60 years of age worldwide have OA, which is characterized mainly by pain and stiffness in the joints, can seriously affect the health and quality of life of patients, and is one of the main causes of disability in adults [3]. Artificial joint replacement is currently considered for patients with advanced OA who are elderly (usually > 65 years) and have severe pain. The treatment of early- to mid-stage OA is not yet effective and is mainly based on symptomatic supportive therapy, such as step analgesia, exercise restriction and rehabilitation training, which can improve patients' quality of life and delay the progression of OA to a certain extent but cannot stop the disease at its root [4–6]. The current approach for conservative treatment of OA constitutes mainly NSAIDs (non-steroid anti-inflammatory drug) and COX-2(Cyclooxygenase-2) inhibitors; however, they only relieve the patient's pain and do not stop the progression of OA, and it is thus important to identify new and better therapeutic drugs.

There is increasing evidence of high expression of inflammatory factors in cartilage tissue, joint fluid and plasma in patients with OA, a low-grade inflammatory joint disease where persistent inflammation is one of the key drivers of articular cartilage degeneration [7–9]. IL-1β is a major inducer of OA and accelerates the degradation of cartilage matrix by stimulating increased expression of extracellular matrix-degrading enzymes and numerous proteoglycanases in chondrocytes. The expression of cartilage-specific proteins such as type II collagen and the abundance of aggregated proteoglycans decrease significantly in response to inflammatory stimuli, accelerating cartilage ageing and degradation and ultimately leading to an imbalance in chondrocyte anabolism and catabolism and even to chondrocyte apoptosis [10]. Thus, the persistence of inflammation with degradation of the extracellular matrix (ECM) may significantly contribute to the pathogenesis of OA.

PI3K/Akt belongs to the serine/threonine protein kinase family and is involved in the regulation of several downstream cellular targets, such as activation of the NF ‐ κB pathway through phosphorylation of IкBα and p65 [11–13]. Furthermore, it has been suggested that cytokine-induced activation of the PI3K signalling pathway is associated with NF-κB-dependent signalling pathways in different cell types [14–16]. Therefore, the PI3K/Akt pathway is considered a potential key target for the treatment of degenerative cartilage lesions.

Cucurbitacin E(CuE) is an oxygenated tetracyclic triterpenoid [17] that has received much attention for its antitumour properties [18–20] (in melanoma, breast tumours, and colon cancer). Additionally, Cucurbitacin E has been reported to have anti-inflammatory properties. For example, a Cucurbitacin isolated from the juice extract of *Ecballium elaterium*, which also contains Cucurbitacin E, exhibits potential anti-inflammatory, analgesic and antipyretic activity in rodents [21]. The crude ethanolic extract of the roots of Cucurbitaceae family members exerts anti-inflammatory and analgesic effects in rats and mice [22]. However, the mechanism of action of Cucurbitacin E in OA chondrocytes remains unclear.

Here, we investigated the inflammatory effects of Cucurbitacin E on IL-1β-stimulated OA chondrocytes compared with its protective effects on chondrocytes, evaluated its protective effects on central cartilage in an in vivo mouse model of DMM-induced OA, and explored its relationship with the PI3K/Akt pathway.

## Materials and methods

### Reagents

Antibodies targeting Matrix metallopeptidase 13 (MMP13),collagen type II(Collagen II), (Interleukin—1β)IL-Iβ, PI3K, and p-PI3K were purchased from ImmunoWay (Texas, USA); antibodies targeting Akt, p-Akt,COX-2 and GAPDH were purchased from Hua'an (Hangzhou, China); and trypsin, radioimmunoprecipitation assay (RIPA) buffer, phenylmethanesulfonyl fluoride (PMSF), a BCA kit, 5 × SDS‒PAGE sample loading buffer, BeyoECL Plus and penicillin–streptomycin were purchased from Beyotime (Shanghai, China). Collagenase II was obtained from Sigma (St. Louis, MO, USA). DMEM/F12 was purchased from HyClone (South Logan, UT, USA). Foetal bovine serum was obtained from Vicente. TRIzol reagent was purchased from Invitrogen (Carlsbad, CA, USA). 5 × HiScript II qRT SuperMix II was purchased from Vazyme (Nanjing, China). Goat anti-rabbit IgG and goat anti-mouse IgG were purchased from ZSGB-BIO (Beijing, China). Recombinant human IL-1β was obtained from PeproTech (Rocky Mount, NJ, USA). Cucurbitacin E and SC79 (Akt activator) were obtained from MedChemExpress (Shanghai, China). A Cell Counting Kit-8 was purchased from 7Sea Biotechnology (Shanghai, China). All gene primers were synthesized by Jereh Biotechnology (Shanghai, China). Safranin O staining solution was purchased from Solarbio (Beijing, China). The reagents for saffron staining, safranin-O/Fast Green staining, Alcian blue staining and HE staining were all obtained from Servicebio (Beijing, China). Polyvinylidene fluoride (PVDF) membranes and dimethyl sulfoxide (DMSO) were purchased from Thermo Fisher Scientific (Shanghai, China).

### Cell culture and animal model

Human cartilage was obtained from OA patients who underwent total knee arthroplasty (TKA) at the First Affiliated Hospital of Anhui Medical University. This study was approved by the clinical Ethics Committee of the First Affiliated Hospital of Anhui Medical University (*Reference number: PJ2023-12-63*). The cartilage pieces were minced in a sterile environment, with 0.25% trypsin added to a 2 × volume of minced cartilage, followed by digestion in a 37 °C incubator for 30 min.After discarding the trypsin, the cartilage was soaked with an equal amount of medium containing 0.1% collagenase II for 24 h. Then, the digested cartilage was centrifuged at 500 r/min for 5 min. We obtained the supernatant from the digested tissues after low-speed centrifugation (500 r/min × 5 min), and the supernatant was centrifuged again (1200 r/min × 5 min) to obtain the chondrocyte precipitate. Isolated chondrocytes were resuspended in DMEM/F-12 (an antibiotic mixture containing 10% FBS, 1% L-glutamine, and 1% penicillin and streptomycin) and incubated with 5% CO_2_ at 37 °C. The experimental mice were C57BL/6 mice obtained from Beijing Spelford Biotechnology Co., Ltd. (purchase batch number: No.110324221100930336). The C57BL/6 mice were 8-week-old male mice. After 1 week of adaptation in specific pathogen-free (SPF)-grade housing in the laboratory, the C57BL/6 mice were randomly divided into three groups (n = 6–8 mice/group): the sham group, DMM group and Cucurbitacin E intervention group. One week later, mice in the three groups were anaesthetized. The mice in the DMM group and Cucurbitacin E intervention group underwent DMM surgery, in which an incision was made in the skin of the knee joint and the medial collateral ligament of the knee joint was cut, while the mice in the sham group underwent sham surgery without cutting of the medial collateral ligament of the knee joint. One month later, Cucurbitacin E dissolved in solvent (0.5 mg/kg) was injected into the joint cavity of each mouse in the Cucurbitacin E intervention group twice a week for 3 months [23–25]. The mice in the other two groups were injected with equal amounts of normal saline in the joint cavity. We assessed the knee joint condition in mice using the OARSI score, a standard used to assess the severity of osteoarthritis. Developed jointly by the European Osteoarthritis Society and the U.S. The Food and Drug Administration, the OARSI is one of the most commonly used clinical criteria for evaluating osteoarthritis. The schematic of the animal experiment is shown in Fig. 5A. This study was approved by the Ethics Committee of the Animal Experimental Center of Anhui Medical University (*Reference number: LLSC20231221*).

### Cell viability assay (CCK-8 assay)

The cytotoxicity of Cucurbitacin E in chondrocytes was evaluated by a Cell Counting Kit 8 (CCK-8) assay. Chondrocytes were spread evenly in 96-well plates (5000 cells/well), incubated in a warm oven for 24 h, and then treated with different concentrations of Cucurbitacin E (1, 5, 10, 20, 40, and 80 nM) for 24 h. Subsequently, 10 μl of CCK-8 solution was added to each well, and the plates were incubated for 2 h at 37 °C. The absorbance at 450 nm was measured.

### Chondrocyte toluidine blue staining and safranin O staining

Chondrocytes were seeded in 12-well plates and incubated for 48 h. The chondrocytes were then washed 3 times with PBS solution and fixed with 4% paraformaldehyde solution for 10 min. Paraformaldehyde was washed away with PBS, and the chondrocytes were observed directly under the electron microscope for imaging; after paraformaldehyde fixation, toluidine blue staining was performed by treatment with 1% toluidine blue solution for 30 min at room temperature followed by the washing of excess toluidine blue dye from the surface with PBS, and the chondrocytes were then observed under the microscope and photographed. Chondrocytes were inoculated in 24-well plates. At confluence, chondrocytes were treated with Cucurbitacin E (10 nM) and IL-1β (10 ng/mL), and the medium was changed every 24 h. At the indicated time points, the chondrocytes were washed with PBS and fixed with 4% paraformaldehyde for 30 min. The chondrocytes were then incubated with safranin O cartilage staining solution (Solabio, China). After incubation at room temperature for 30 min, the staining solution was removed, and the chondrocytes were washed with PBS. Staining was photographed.

### Drug preparation and incubation

Cucurbitacin E in powder form was dissolved in DMSO, and different stock solutions used to prepare the working solutions of the desired drug concentrations (1 mM, 5 mM, 10 mM) were prepared. The IL-1β concentration was adjusted to 10 µg/ml with exclusive diluent. Chondrocytes were seeded in six-well plates and incubated for 48 h. The premade stock solutions of different concentrations of Cucurbitacin E were diluted with DMEM/F12 to the desired concentrations (1 nM, 5 nM, 10 nM), and chondrocytes then were incubated with various concentrations of Cucurbitacin E. After a 2 h pretreatment, IL-1β was added at a diluted concentration of 10 ng/ml to mimic the induction of osteoarthritic properties in cells in vitro, and the cells were then incubated for 24 h. The chondrocytes were incubated with various concentrations of Cucurbitacin E. SC79 was dissolved in DMSO, and the concentration of DMSO in all experimental groups was lower than 0.1%. The chondrocytes in the treatment group were pretreated with SC79 (4 µg/ml) for 1 h to activate the Akt pathway [26].

### Western blotting

The expression of target proteins was measured by immunoblotting. For immunoblotting, Chondrocytes were lysed with a mixture of radioimmunoprecipitation assay (RIPA) buffer and phenylmethanesulfonyl fluoride (PMSF) (100:1), and the protein concentrations were measured using a BCA kit after purification of total protein by high-speed cryogenic centrifugation (12,000 g/min × 15 min, 4 °C). Then, we mixed the supernatant with 5 × SDS‒PAGE sample loading buffer. Equal amounts of protein were separated by SDS‒PAGE using a 10% or 12% polyacrylamide gel. The proteins were then transferred onto PVDF membranes, which were placed in Tris-buffered saline with Tween 20 (TBST)-5% skim milk for 2 h at room temperature. After washing, the membranes were incubated with the corresponding antibody specific for IL-1β (1:1000), COX-2 (1:1000), MMP-13 (1:1000), Collagen II (1:1000), PI3K (1:1000), p-PI3K (1:1000), Akt (1:1000), p-Akt (1:1000), or GAPDH (1:1000) at 4 °C overnight. The membranes were then washed at room temperature and incubated in TBST-5% skim milk containing goat anti-rabbit IgG or goat anti-mouse IgG for 2 h. The membranes were washed again with TBST, and signals were detected using BeyoECL Plus.

### RT‒qPCR

Chondrocytes were pretreated with Cucurbitacin E (1, 5, 10 nM) for 2 h and stimulated with or without IL-1β (10 ng/ml) for 24 h, and total RNA was then extracted. TRIzol reagent was used according to the manufacturer’s instructions, and cDNA was reverse transcribed according to the mRNA concentration. The Agilent Mx3000P system was used for quantitative real-time PCR under the following thermal cycling conditions: predenaturation at 95 °C for 5 min, followed by 40 cycles of denaturation at 95 °C for 10 s, annealing at 60 °C for 30 s, denaturation at 95 °C for 15 s, annealing at 60 °C for 30 s, and denaturation at 95 °C for 15 s. The primers were designed with the help of the NCBI Primer-BLAST tool. The primer sequences are listed as follows: MMP13(human): *(Forward) 5′CCTTGATGCCATTACCAGTCTCC3′, (Reverse) 5′AAACAGCTCCGCATCAACCTGC3′*; Collagen II (human): *(Forward) 5′CCTGGCAAAGATGGTGAGACAG3′, (Reverse) 5′CCTGGTTTTCCACCTTCACCTG3′*; GAPDH (human): *(Forward) 5′ACCCAGAAGACTGTGGATGG3′, (Reverse) 5′TTCAGCTCAGGGATGACCTT3′.*

### Fluorescence microscopy

Chondrocytes were seeded in 12-well plates with coverslips for culture, cotreated with IL-1β (10 ng/ml) or IL-1β in combination with 10 nM Cucurbitacin E, and incubated with serum-free medium for 24 h. Next, the coverslips were removed. After being washed three times with phosphate-buffered saline (PBS), the chondrocytes were fixed with 4% paraformaldehyde for 30 min at room temperature, washed three times with PBS, and permeabilized with 0.5% Triton X-100 for 20 min at room temperature. Then, the cells were blocked with 5% BSA at 37 °C, washed with PBS and incubated with primary antibodies against MMP13 (1:200) and Collagen II (1:200) for 24 h at 4 °C. Subsequently, the cells were washed three times with PBS and incubated with goat anti-rabbit IgG for 2 h at room temperature. Then, nuclei were labelled with 4',6-diamidino-2-phenylindole (DAPI) for 10 min. The slides were blocked after washing with PBS and imaged with a fluorescence microscope.

### Bioinformatics analysis and screening

First, the NCBI website (https://www.ncbi.nlm.nih.gov/) was used to download osteoarthritis and related normal cartilage gene expression data from the GSE114007 and GSE117999 datasets in the GEO database. The data in the GSE114007 dataset [27] are based on gene sequencing with the GPL11154 platform and constitute the gene expression data of 18 normal cartilage tissues and 20 OA cartilage tissues. The data in the GSE117999 dataset are based on gene sequencing with the GPL20844 platform and constitute the gene expression data of 10 normal cartilage tissues and 10 OA cartilage tissues. Then, a Perl script was used to combine the GSE114007 and GSE117999 datasets. To reduce batch effects between the two sets of data and errors in screening differential genes, the normalizeBetweenArrays function of R software was used to normalize the combined data. At the same time, the R software limma package was used for analysis of differentially expressed genes in normal samples and OA samples. In this analysis, |logFC|> 0.585 was set as the screening criterion for differences in expression, and p < 0.05 was considered to indicate a significant difference. Finally, KEGG pathway enrichment analysis of the significantly differentially expressed genes was performed by the "ClusterProfiler" package in R, and p < 0.05 was set as the criterion for significance (R version R4.1.2).

### Cellular thermal shift assay

CETSA is an intracellular assay to measure the binding efficiency of a drug to a target protein and is based on the principle that the target protein generally becomes stable when bound to a drug molecule. That is, with increasing temperature, the protein is degraded, and when the protein binds to the drug, the amount of undegraded protein increases at the same temperature [28, 29]. In as accordance with the cell culture method described above, human primary chondrocytes were cultured in a large 10 cm dish, and RIPA buffer containing a protease inhibitor was added for lysis on ice for 20 min; the lysates were then transferred to 1.5 mL EP tubes and centrifuged at 4 °C for 15 min at 12,000 r/min, and each tube was then thoroughly shaken and divided into 2 tubes. Cucurbitacin E at the working concentration was added to 1 tube, and an equal volume of DMSO solution was added to the other tube; the tubes were shaken well and incubated at room temperature for 2 h. Then, the two tubes of solution were evenly divided into 5 small EP tubes, and each tube was incubated at a different temperature gradient set for 5 min. Finally, the supernatant was centrifuged, mixed with SDS solution, and heated in a metal water bath at 95 °C for 10 min. Western blot analysis was performed after these steps were completed.

### Molecular docking model and molecular dynamics simulation

The 3D molecular structure of the compound Cucurbitacin E (ID:5281319) was downloaded from the PubChem website (https://pubchem.ncbi.nlm.nih.gov/) in sdf format. Then, the Cucurbitacin E structure was optimized and assigned a partial charge under the Amber10-EHT force field (MOE2022). Then, 81 poses of the optimized structure were generated using the *conformation search module* of MOE for molecular docking. The three-dimensional structure was retrieved from the Protein Data Bank (https://www.rcsb.org/structure/3LJ3). The crystal model (3LJ3) was protonated and optimized by the MOE plugin “*QuickPrep*”. In the docking process, the coarse-grained model and the side chains of the contact residues were optimized, up to 1000 conformations were retained using the London δ scoring function and were then docked with IFD (induced fit docking) and optimized using energy minimization, and the binding energies were calculated by the GBVI/WSA scoring function. Finally, the top 100 docking poses were retained and clustered, and the best pose was selected as the final pose.

The complex structures were optimized using the *Protein Preparation Wizard* [30] panel (Schrödinger 2021) by correcting the bond order, adding hydrogen atoms, distributing charges, and predicting protonation states (pH 7.0). The OPLS4 force field was used for constrained energy optimization to eliminate atomic conflicts in the structure with the RMSD of heavy atoms converging to 0.3 Å, and the side chain position was optimized to obtain a reasonable side chain structure; the resulting structures were saved as Protein-F6724-4856.pdb (Cpd1), Protein-STOCK1N-88112.pdb (Cpd2), and Protein-STOCK1N-90146.pdb (Cpd3). We employed the *Desmond* [31] program for molecular dynamics simulation based on the OPLS4 force field. The complexes were solvated in a cubic box with the TIP3P water models, and NaCl (0.15 mol/L) was added to neutralize the system (Additional file 1: Fig. S1A). The system was subjected to minimization and equilibration for 100 ps (10,000 steps with Brownian motion simulation) to adequately equilibrate complexes and solvent molecules. Harmonic position restraints were applied on the backbone of the protein with a force constant of 1 kcal mol^−1^ Å^−2^ and a time step of 2 fs. An MD simulation with equilibration for 100 ns was performed with the NPT ensemble at 300 K with a Nose–Hoover chain thermostat (relaxation time 100 ps) and at 1 atm with an isotropic Martyna–Tobias–Klein barostat (relaxation time 100 ps). The time step was set to 2.0 fs. Short-range electrostatic interactions were calculated with a cut-off of 9.0 Å, and long-range electrostatic interactions were calculated with the PME method. The trajectories were saved every 5 ps for further analyses.

### Immunohistochemical analysis and tissue staining

Specimens were fixed with 4% paraformaldehyde for 24 h and decalcified with 10% ethylenediaminetetraacetic acid for 4 weeks. The tissues were embedded in paraffin and sliced perpendicular to the articular cartilage surface into 5-μm-thick sections, which were soaked in three different dye solutions for staining: saffron O/solid green, HE, and Alcian blue [32]. The extent of cartilage degeneration was assessed using the Osteoarthritis Research Society International (OARSI) cartilage histopathological assessment system as follows (six OA grades): 0 = intact surface; 1 = intact cartilage; 2 = discontinuous surface; 3 = vertical fissures; 4 = erosion; 5 = denudation; and 6 = deformation.

For immunohistochemical staining, tissue sections were dewaxed in xylene solutions, and 3% hydrogen peroxide was then used to quench endogenous peroxidase activity. The sections were incubated with 0.4% pepsin (Sigma‒Aldrich) in 1 mM hydrochloric acid at 37 °C for 1 h for antigen repair. The blocking solution was a PBS solution containing 5% BSA. After incubation with blocking solution at 37 °C for 30 min, the sections were incubated with primary antibodies against MMP13 (1:200),Collagen II (1:200), IL-1β (1:100), and COX-2 (1:100). Subsequently, counterstaining was performed with haematoxylin. Images of the selected areas were acquired using an optical microscope, and expression was quantified from the images based on histological staining.

### Statistical analysis

All data are expressed as the means ± standard deviations. One-way analysis of variance (ANOVA) with SPSS V.23.0 (SPSS Inc., Chicago, USA) was used to analyse differences among groups. P < 0.05 was considered to indicate a statistically significant difference.

## Results

### Identification of human chondrocytes

**Fig. 1:**
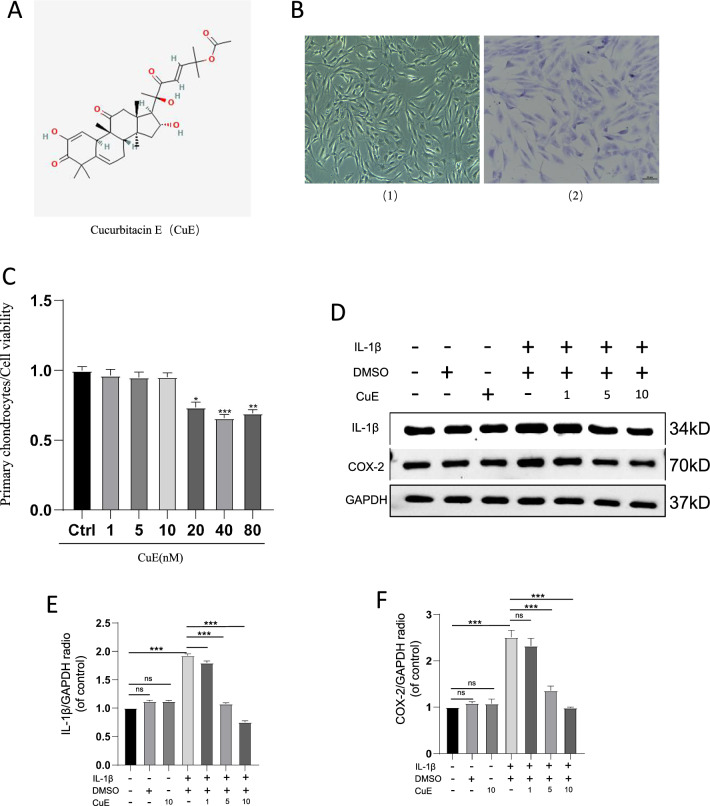
Cucurbitacin E alleviated IL-1β-induced inflammation. **A** Structural formula of Cucurbitacin E; **B** Morphological structure of human chondrocytes; B (1) shows the field of view under an optical microscope, and B (2) shows toluidine blue staining of chondrocytes. The scale bar represents 50 µm; **C** Activity of Cucurbitacin E at different concentrations in chondrocytes. **D** Western blot analysis of the expression of IL-1β and COX-2 in inflamed chondrocytes stimulated with IL-1β and treated with Cucurbitacin E at different concentrations (1, 5, and 10 nM). **E** and **F** Quantitative analysis of IL-1β and COX-2 protein expression (n = 3). The significance of differences between groups is expressed as P values (***P < 0.001, **P < 0.01, *P < 0.05,^ns^P > 0.05)

The chemical structure of Cucurbitacin E is shown in Fig. 1A. To identify isolated human primary chondrocytes, chondrocytes were observed by bright field microscopy [Fig. 1B(1)]. As shown in Fig. 1B(2), toluidine blue can stain proteoglycans in the cytoplasm of chondrocytes purple, and the images show that human primary chondrocytes are spindle shaped and elliptical/spindle shaped.

### Cucurbitacin E viability assay on chondrocytes

We tested the effect of different concentrations of Cucurbitacin E on the viability of human primary chondrocytes. As shown in Fig. 1C, the CCK-8 assay results showed that Cucurbitacin E treatment of human chondrocytes for 24 h was safe at concentrations below 20 nM; thus, we chose concentrations of Cucurbitacin E within the range of safe concentrations, i.e., 1, 5 and 10 nM, for subsequent experiments.

### Cucurbitacin E decreases the expression of inflammatory factors induced by IL-1β in human chondrocytes

Inflammation is thought to be an important mechanism in the development of OA; thus, we examined the expression of IL-1β-induced inflammatory factors in chondrocytes by Western blot analysis, as shown in Fig. 1D-F. The expression of inflammatory factors, including IL-1β and COX-2, was significantly increased in IL-1β-stimulated chondrocytes compared with chondrocytes not treated with IL-1β, while Cucurbitacin E significantly inhibited the expression of inflammatory factors in a dose-dependent manner. In contrast, neither DMSO alone nor DMSO/Cucurbitacin E inhibited the expression of IL-1β and COX-2. These data suggest that under pathological conditions, Cucurbitacin E can exhibit anti-inflammatory activity by inhibiting the expression of inflammatory factors in OA chondrocytes.

### Cucurbitacin E alleviates the upregulation of MMP13 expression and the downregulation of Collagen II expression in chondrocytes caused by IL-1β stimulation

**Fig. 2:**
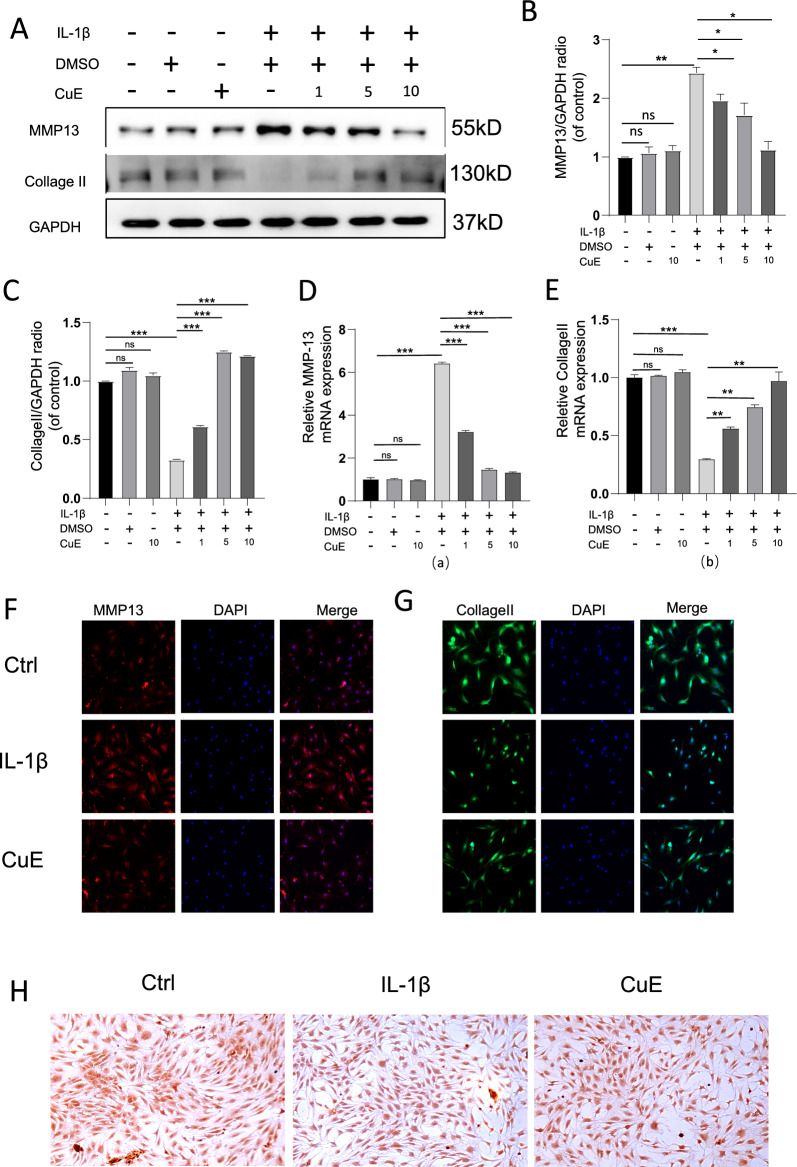
Cucurbitacin E decreased the high expression of MMP13 and increased the low expression of Collagen II in IL-1β-stimulated osteoarthritis chondrocytes. **A** Figure showing the expression of MMP13 and Collagen II in IL-1β-induced osteoarthritis chondrocytes treated with different concentrations of Cucurbitacin E (1, 5, 10 nM), as measured by Western blotting. **B** and **C** Quantitative analysis of MMP13 and Collagen II protein expression, respectively (n = 3). **D** and **E **The expression of MMP13 and Collagen II in IL-1β-induced osteoarthritis chondrocytes treated with different concentrations of Cucurbitacin E (1, 5, 10 nM) was measured by qPCR. Immunofluorescence images of chondrocytes under different treatment conditions (Ctrl, IL-1β, Cucurbitacin E) are shown in **F** and **G**. **H** Diagram showing safranin O staining of chondrocytes under different treatment conditions (Ctrl, IL-1β, CuE). The significance of differences between groups is expressed as P values (***P < 0.001, **P < 0.01, *P < 0.05,^ns^P > 0.05)

**Fig. 3:**
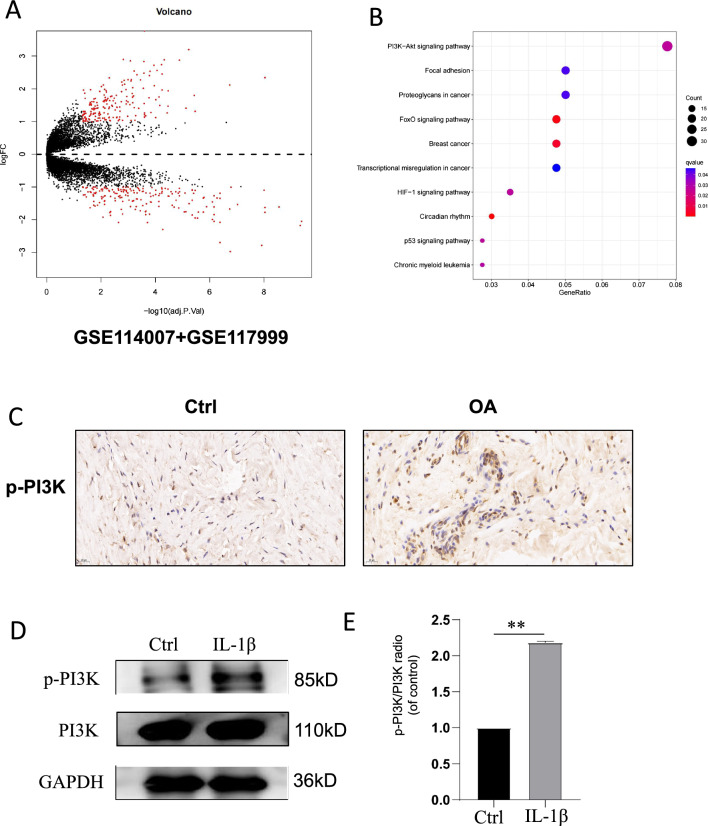
The PI3K/Akt pathway is activated in osteoarthritis chondrocytes. **A** Volcano plots of differentially expressed genes in normal and osteoarthritic cartilage based on the GSE114007 and GSE117999 datasets obtained from the GEO database. **B** KEGG enrichment analysis of differentially expressed genes in osteoarthritic cartilage. **C** Immunohistochemical staining of the p-PI3K protein in normal and osteoarthritic cartilage. **D** Western blotting was used to examine the activation of PI3K in IL-1β-stimulated osteoarthritis chondrocytes. **E** ImageJ software was used to analyse the activation of the PI3K protein. The significance of differences between groups is expressed as P values (**P < 0.01)

Collagen II expression was inhibited by IL-1β treatment in chondrocytes, whereas Western blot analysis (Fig. 2A–C) and RT‒qPCR (Fig. 2D, E) showed that IL-1β treatment of chondrocytes significantly upregulated the expression of MMP13 while decreasing Collagen II expression and that these changes were alleviated by Cucurbitacin E treatment, which reduced the expression of MMP13 and increased the expression of Collagen II in a dose-dependent manner. We evaluated the expression of MMP13 and Collagen II in IL-1β-stimulated chondrocytes by immunofluorescence staining (Fig. 2F–G), and the results showed that the expression level of MMP13 in IL-1β-stimulated chondrocytes was higher than that in the unstimulated group, while the expression level of Collagen II in IL-1β-stimulated chondrocytes was lower than that in the unstimulated group. However, after coculture with Cucurbitacin E, the expression level of MMP13 in chondrocytes was lower than that in the stimulated group, and the expression level of Collagen II in chondrocytes was higher than that in the stimulated group. In addition, we found no effect on Collagen II expression in the DMSO alone group compared with the DMSO/Cucurbitacin E group. In addition, we found that the aggregated proteoglycans of the extracellular matrix were stained by safranin O and that the IL-1β-induced loss of aggregated proteoglycans in chondrocytes was mitigated by coculture with Cucurbitacin E (10 nM) compared to that in normal chondrocytes (Fig. 3H). These results suggest that Cucurbitacin E may be a potent inhibitor of IL-1β-triggered expression of extracellular matrix degradation-related proteins in chondrocytes.

### PI3K/Akt pathway proteins are highly expressed in osteoarthritic cartilage

First, we used the ‘limma’ R package to analyse microarray data from the OA-related GSE114007 and GSE117999 datasets (|logFC|> 0.585, P < 0.05). A volcano plot (Fig. 3A) was generated using Bioconductor. KEGG analyses were conducted with a P value of < 0.05 as the cutoff value for significant enrichment, and the PI3K/Akt signalling pathway was identified (Fig. 3B). We extracted cartilage tissues from normal individuals and patients with osteoarthritis and conducted immunohistochemical analysis. It was found that the level of p-PI3K was higher in cartilage tissues of patients with osteoarthritis than in normal tissues (Fig. 3C). In addition, we demonstrated this pattern in human osteoarthritis chondrocytes stimulated with IL-1β; that is, the p-PI3K level was high in osteoarthritis chondrocytes (Fig. 3D). These results demonstrate activation of the PI3K/Akt pathway in chondrocytes stimulated with IL-1β.

### Cucurbitacin E can bind the PI3K protein to enhance its thermal stability

**Fig. 4:**
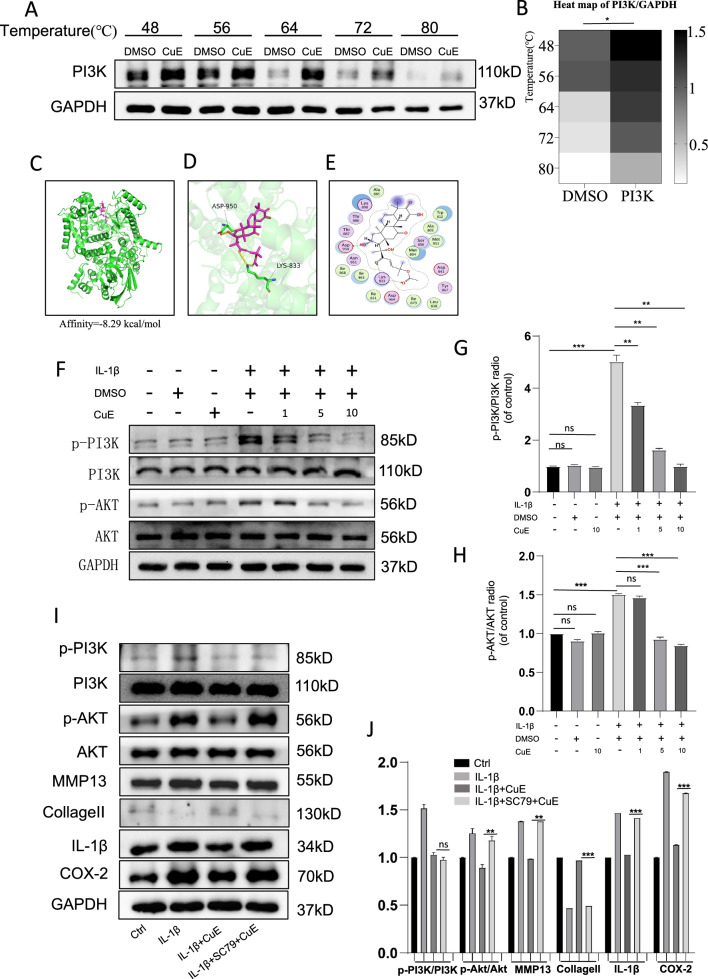
Cucurbitacin E can bind to the PI3K protein and inhibit its phosphorylation. **A** Cellular thermal shift assays of the PI3K protein. **B** Heatmap showing the results of the cellular thermal shift assays of the PI3K protein; the darker the colour, the higher the protein expression level. **C** Global view of interacting proteins and molecules: Cucurbitacin E–PI3K docking conformation (affinity = − 8.29 kcal/mol). **D** 3D interaction local view: the PI3K protein secondary structure is shown in the green cartoon, the ligand carbon atoms are shown in magenta, oxygen atoms are shown in red, nitrogen atoms are shown in blue, and the yellow dashed lines indicate hydrogen bonds. Cucurbitacin E formed hydrogen bonds with Lys833 and Asp950 in the protein. **E** 2D interaction view, with green arrows indicating hydrogen bonds. **F** Western blotting was performed to measure the expression of PI3K/Akt pathway proteins in IL-1β-stimulated osteoarthritis chondrocytes treated with different concentrations of Cucurbitacin E (1, 5, and 10 nM). **G** and **H** The expression of the PI3K/Akt pathway proteins in Figure F was analysed by ImageJ software. **I** and **J** SC79 was used to investigate the effect of CuE on IL-1β-stimulated chondrocytes, and ImageJ software was used for quantitative analysis of protein levels. The significance of differences between groups is expressed as P values (***P < 0.001, **P < 0.01, *P < 0.05,^ns^P > 0.05)

We measured the thermal stability of the PI3K protein at different temperatures. With increasing temperature, the abundance of the PI3K protein gradually decreased (Fig. 4A), but the abundance of the PI3K protein in the Cucurbitacin E group was higher than that in the DMSO group at the same temperature (Fig. 4B), indicating that Cucurbitacin E increased the thermal stability of the PI3K protein. Cucurbitacin E can effectively bind the PI3K protein.

### Molecular docking of Cucurbitacin E and PI3K and the corresponding molecular dynamics simulation

The affinity between Cucurbitacin E and the inhibitory sites in the PI3K protein was analysed by molecular docking. In this analysis, the chemical structure of Cucurbitacin E was used, as shown in Fig. 1A. By examining all the generated models, we found that Cucurbitacin E interacts with and docks at the inhibitory site of PI3K, and macro and local horizontal views of these interactions are shown as ribbon models (Fig. 4C). A high affinity (− 8.29 kcal/mol) was observed for hydrogen bonding between Lys833 and Asp950 in Cucurbitacin E and PI3K (Fig. 4D,E).

Statistical analysis of the RMSD and RMSF values calculated from the molecular dynamics simulation showed that the structural system began to converge after approximately 15 ns and that the RMSD eventually fluctuated between 2.0 and 2.5 Å (Additional file 1: Fig. S1B). The RMSF value of the main structure, except the head and end groups and part of the loop structure, fluctuated very little, approximately 1 Å (Additional file 1: Fig. S1C). The physical and chemical properties of the ligand (CuE) remained stable throughout the simulation (Additional file 1: Fig. S1D). The structure trajectory between 60 and 100 ns with a simulated trajectory balance was selected for MMGBSA analysis combined with free energy analysis, and the script thermal_mmgbsa.py was used for calculation. Initially, one structure was selected every 20 frames, and the properties of 200 structures were calculated by sampling each protein‒protein trajectory. The statistical results are shown in Additional file 1: Fig. S1E. The average value was − 55.60 kcal/mol. Amino acid analysis of the simulated trajectory revealed that Ala805, Ser806, Lys833, Asp950 and Asn951 are the five key amino acids contributing to hydrogen bonding and that Lys833 and Asp964 can form a water bridge with the ligand (Additional file 1: Fig. S1F). Structure clustering was performed on the structure of the simulated trajectory between 60 and 100 ns, and a representative structure was selected for comparison with the original structure. As shown in Additional file 1: Fig. S1G, the secondary structure of the protein hardly changed, and the structure of the ligand maintained a consistent binding conformation without major changes. Through structural stability analysis, free energy analysis, key amino acid analysis and binding conformation analysis, the binding between CuE and PI3K was shown to be stable.

In conclusion, Cucurbitacin E may inhibit the development of OA by interacting with PI3K.

### Cucurbitacin E inhibits IL-1β-induced abnormal activation of the PI3K/Akt signalling pathway in human chondrocytes

Next, we sought to investigate the mechanisms by which Cucurbitacin E exhibits anti-stimulatory chondroprotective effects. Recent literature suggests that the inflammation and MMP expression observed in OA pathogenesis are associated with PI3K/Akt pathway activation. The cellular thermal shift assay results proved that Cucurbitacin E can bind to the PI3K protein, and the molecular docking results showed that Cucurbitacin E binds tightly to the inhibitory pocket of PI3K. Therefore, we investigated whether Cucurbitacin E exerts its chondroprotective effects by inhibiting the PI3K/Akt signalling pathway in IL-1β-stimulated human OA chondrocytes. The data suggest that IL-1β stimulation activates the phosphorylation of PI3K and Akt. Treatment of OA chondrocytes with Cucurbitacin E inhibited the expression and phosphorylation of PI3K and Akt in a dose-dependent manner (Fig. 4F–H). At the same time, we performed a reciprocal validation experiment by preactivating Akt with SC79 (4 µg/ml) and showed that the effect of Cucurbitacin E on IL-1β-induced chondrocyte degeneration was reduced or abolished (Fig. 4I–J). Overall, these findings indicate that Cucurbitacin E may exert anti-inflammatory effects and inhibit the expression of MMPs by inhibiting IL-1β-induced PI3K/Akt signalling.

### Cucurbitacin E inhibits cartilage degradation in DMM model mice

**Fig. 5:**
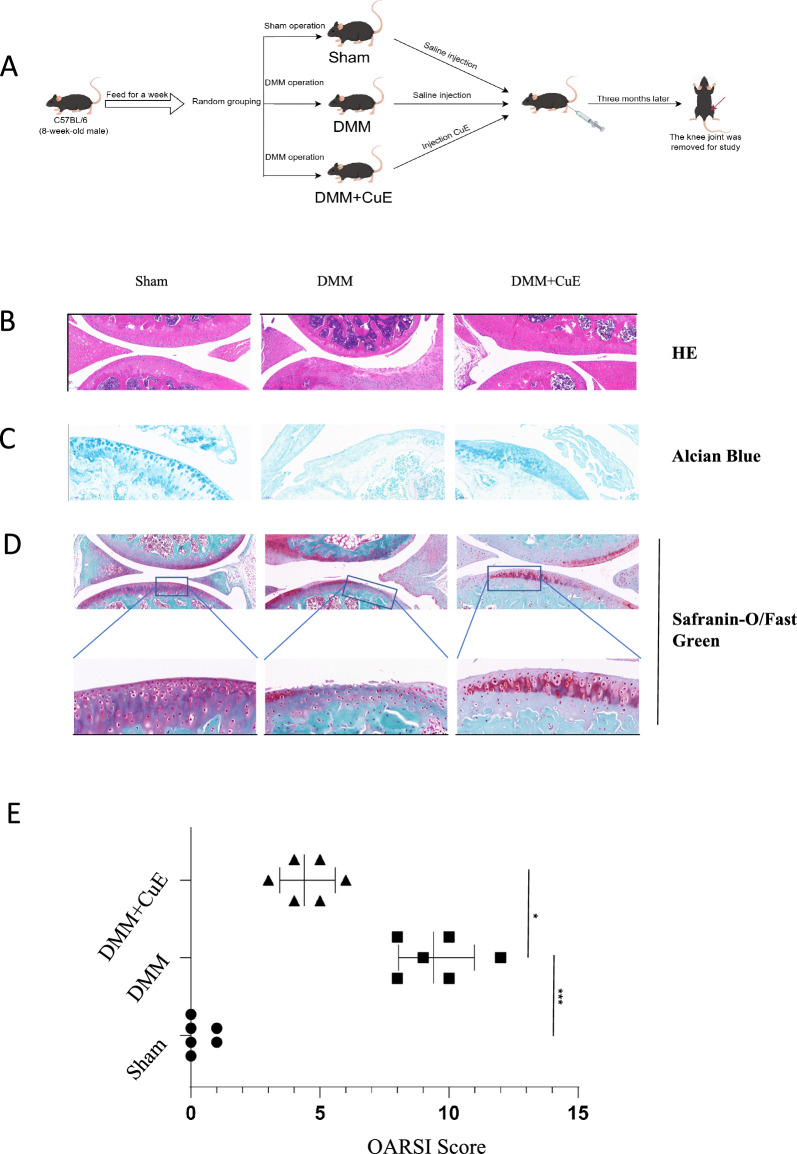
Cucurbitacin E inhibits cartilage degradation in DMM model mice. **A** Animal experimental roadmap. **B** HE staining of cartilage from the three groups (scale bar, 100 µm); **C** Alcian blue staining of cartilage from the three groups (scale bar, 50 µm); **D** Safranin O/Fast Green staining (scale bar, 100 µm) and local magnification (scale bar, 50 µm) of cartilage from the three groups. **E** OARSI scores of cartilage in different groups (normal control group, DMM group, DMM + Cucurbitacin E group). The significance of differences between groups is expressed as P values (***P < 0.001, **P < 0.01, *P < 0.05,^ns^P > 0.05)

To assess whether Cucurbitacin E has a protective effect on OA progression in vivo, we performed surgical procedures on C57BL/6 mice to establish a mouse model of OA by destabilizing the medial meniscus. Details are provided in the experimental methods section, and the experimental strategy is shown in Fig. 5A. We performed histological analysis by staining the knee cartilage tissue of mice with HE (Fig. 5B), Alcian blue (Fig. 5C), and safranin-O/Fast Green (Fig. 5D) and scored OA in each group by the OARSI standard. Alcian blue stained the periphery of the knee cartilage of C57BL/6 mice a vivid blue, as shown in Fig. 5C. The staining in the knee joints of C57BL/6 mice in the DMM group was light, indicating that the knee joint cartilage of C57BL/6 mice in the DMM group was lost and uneven, with damage and wear. The staining around the cartilage in the Cucurbitacin E intervention group was stronger than that in the DMM group, and the edge was relatively smooth. HE stained the cartilage of C57BL/6 mice red, and cartilage destruction in the C57BL/6 mice in each group was clearly visible. As shown in Fig. 5B, the cartilage edge in C57BL/6 mice in the sham group was smooth and even, while the cartilage edge in C57BL/6 mice in the DMM group was rough. The cartilage of C57BL/6 mice was lost and damaged, but the cartilage edge in mice in the Cucurbitacin E group was smoother than that of mice in the DMM group. By safranin-O/Fast Green staining, the cartilage matrix was visualized as uniformly red and the subchondral bone as green, and the cartilage tissue contrasted sharply with the bone tissue. The results of safranin-O/Fast Green staining in the knee joints in the three groups of mice also confirmed the results of the other two types of staining. Then, we determined the OARSI scores of the three groups of knee joints, and the scoring results are shown in Fig. 5E. The three groups of knee joint tissues were compared by three staining methods, all of which proved that the knee joint cartilage of mice exhibited different degrees of damage after DMM, indicating that the osteoarthritis model was successfully established, and the cartilage damage in the Cucurbitacin E intervention group was attenuated compared to that in the DMM group. This demonstrated that Cucurbitacin E could slow the degeneration of knee cartilage in C57BL/6 mice after DMM.

### Effect of Cucurbitacin E on the expression of IL-1β, COX-2, MMP13, and Collagen II in articular cartilage in the model of DMM-induced OA

**Fig. 6:**
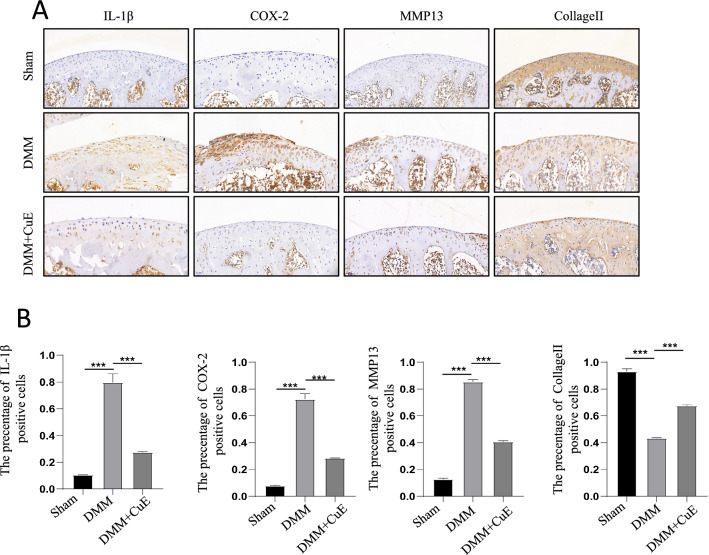
Cucurbitacin E alleviated cartilage degeneration in DMM mice. **A** Immunohistochemical staining of cartilage from the different groups (normal, DMM, DMM + Cucurbitacin E): IL-1β, IL-6, MMP13, and Collagen II. **B** Quantitative analysis corresponding to each immunohistochemical image for IL-1β, COX-2, MMP13 and Collagen II in C57BL/6 model mice, as determined by ImageJ software. The significance of differences between groups is expressed as P values (***P < 0.001, **P < 0.01, *P < 0.05,^ns^P > 0.05)

To demonstrate the effects of Cucurbitacin E in vivo, we performed immunohistochemical staining to evaluate the expression of IL-1β, COX-2, MMP13 and Collagen II in the OA model. The results of immunohistochemical staining (Fig. 6A, B) showed that the expression of Collagen II was lower in the DMM group than in the Sham group and that the expression of IL-1β, COX-2 and MMP13 was elevated in the DMM group compared with the sham group. In contrast, the expression levels of IL-1β, COX-2 and MMP13 were decreased after Cucurbitacin E intervention relative to those in the DMM group, while the expression of Collagen II was restored. These results showed that the expression of ECM-degrading enzymes decreased and collagenase II expression increased after Cucurbitacin E intervention in the model group. In conclusion, Cucurbitacin E treatment delayed the development of OA.

## Discussion

Currently, NSAIDs are the main drugs for the treatment of early OA, but NSAIDs can only control the symptoms of OA and cannot effectively slow the progression of OA, and these drugs have certain side effects [33, 34]. Therefore, it is necessary to explore other potential drugs for the treatment of OA. Inflammation is always associated with the pathogenesis of OA and OA-related symptoms [35]. Studies have shown that persistent inflammation and ECM degradation promote the development of osteoarthritis [36]. Cucurbitacin E is a compound isolated from plants in the Cucurbitaceae family that has been shown to have anti-inflammatory effects [21, 22], but its role and mechanism in osteoarthritic chondrocytes are unclear. In this study, we found that Cucurbitacin E was able to inhibit IL-1β-induced chondrocyte inflammation and ECM degradation.

**Fig. 7:**
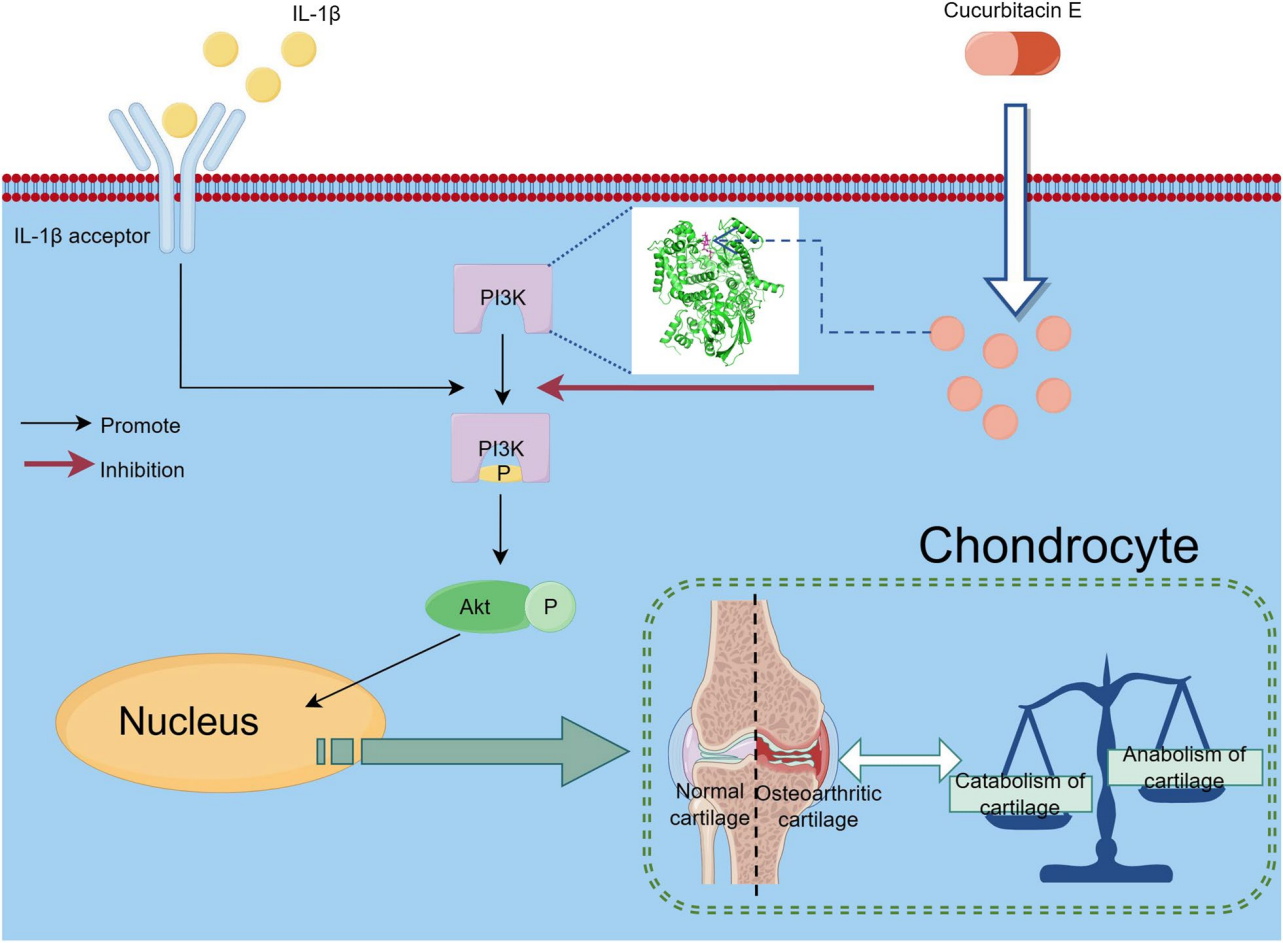
The mechanism by which Cucurbitacin E alleviates IL-1β-induced chondrocyte inflammation and cartilage degeneration by inhibiting the PI3K/Akt pathway (generated with Figdraw)

To further investigate the mechanism of Cucurbitacin E in alleviating cartilage inflammation and degeneration, we analysed the GSE114007 and GSE117999 datasets, which contain gene expression data related to osteoarthritic cartilage and normal cartilage and were downloaded from the GEO database via the NCBI website. Through KEGG pathway enrichment analysis of the significantly differentially expressed genes between normal and osteoarthritic cartilage, it was found that the PI3K/Akt pathway was highly activated in osteoarthritic cartilage. This finding was confirmed at the histological and cellular levels. Previous studies have shown that the PI3K/Akt pathway is closely related to osteoarthritis and that IL-1β can trigger a strong inflammatory response by activating a complex network of signalling pathways, in which PI3K/Akt signalling is closely intertwined with IL-1β-induced inflammation [37]. A large number of studies have shown that PI3K and Akt are rapidly phosphorylated under stimulation with IL-1β [38, 39], suggesting that the PI3K/Akt pathway may mediate the initiation of the inflammatory response. PI3K inhibition is considered to be an anti-inflammatory therapeutic strategy [40]. Human cells express three classes of PI3K catalytic subunits, and the PI3K-Akt pathway is specifically associated with the class I catalytic subunits. Mammals express four class I catalytic subunits (p110α, β, δ, and γ), which encode PIK3CA, PIK3CB, PIK3CD, and PIK3CG, respectively, all of which phosphorylate PIP2 to generate PIP3 [41]. In addition, there is an inhibitory pocket in PI3K, and when a ligand is bound to the inhibitory pocket in PI3K, it can inhibit the phosphorylation of PI3K [38, 42], thus inhibiting the activation of the downstream mediator Akt. We found through cellular thermal shift assays that Cucurbitacin E can effectively bind to PI3K, and we then conducted molecular docking analysis of Cucurbitacin E and the PI3K protein, finding that Cucurbitacin E can effectively bind to the inhibitory pocket of PI3K; in addition, the binding stability of Cucurbitacin E and PI3K was proven by molecular dynamics simulation. We then demonstrated in vitro that Cucurbitacin E inhibits IL-1β-induced PI3K/Akt pathway activation. Combining the results of the above experiments, we hypothesized that Cucurbitacin E prevents the phosphorylation of PI3K by stably binding to the inhibitory pocket of PI3K. To support this hypothesis, we conducted a reciprocal verification experiment. We activated Akt downstream of PI3K by treatment with SC79 and found that Cucurbitacin E lost its effect on alleviating IL-1β-induced inflammation and ECM degradation. These results confirmed our hypothesis that Cucurbitacin E inhibits IL-1β-induced chondrocyte inflammation and cartilage degeneration in osteoarthritis by inhibiting the PI3K/Akt pathway. A schematic diagram outlining the scientific hypothesis is shown in Fig. 7.

We further verified the effect of Cucurbitacin E on the knee cartilage of C57BL/6 mice in vivo. DMM mouse models are widely used to simulate human OA [43, 44] and have been shown to be useful for evaluating drug effectiveness. In this study, calcification, bone sclerosis, chondrocyte loss and matrix degradation were observed in the DMM model mice, and Cucurbitacin E alleviated these pathological changes to a certain extent. Studies have shown that Cucurbitacin E has the potential to slow the progression of OA in vivo.

In addition, this study has some limitations, such as the lack of specific mechanistic studies in vivo and lack of exploration of downstream targets of the PI3K/Akt pathway in vivo. We have learned from previous studies that the NF-κB pathway plays an important role in the inflammatory response and is related to the occurrence and development of OA [45, 46]. More importantly, activation of the PI3K/Akt pathway leads to activation of the downstream NF-κB pathway [47, 48], leading to phosphorylation of iκBα and p65, followed by translocation of the NF-κB complex to the nucleus, where p65 promotes proinflammatory mRNA expression. Therefore, exploration of whether Cucurbitacin E inhibits the PI3K/Akt/NF-KB pathway or other pathways is needed in the future.

In summary, we found that Cucurbitacin E alleviates IL-1β-induced chondrocyte inflammation and cartilage degeneration by inhibiting the PI3K/Akt pathway. In addition, in vivo experiments in mice confirmed that Cucurbitacin E can alleviate the development of OA to a certain extent; thus, Cucurbitacin E can potentially be used as a drug to treat OA.

## Conclusion

Our study showed that Cucurbitacin E attenuated IL-1β-induced chondrocyte inflammation and cartilage degeneration by inhibiting the PI3K/Akt pathway.

### Supplementary Information

**Additional file 1: Figure S1.** Molecular dynamics simulation of Cucurbitacin E and PI3K. **A** The protein is embedded in cubes in the solvent model, the red origin represents the oxygen atom in the water molecule, and the protein is shown in cartoon mode; **B** Molecular dynamics simulation results. Protein stability analysis, protein main chain RMSD values. **C** protein main chain RMSF values. **D** Molecular dynamics simulation results corresponding to the ligand stability analysis: ligand RMSD, ligand RMSF, solvent accessible surface area (SASA), cyclotron radius (RGR). **E** MMGBSA binding free energy analysis. **F** frequency statistics of interacting amino acids; **G** the initial structure (green) and the representative structure (cyan blue) are superimposed.

## Data Availability

Not applicable.
