# Supplementary material for: Cucurbitacin E reduces IL-1β-induced inflammation and cartilage degeneration by inhibiting the PI3K/Akt pathway in osteoarthritic chondrocytes
Source: J Transl Med. 2023 Dec 4;21:880. doi: 10.1186/s12967-023-04771-7 (PMC10696753; doi:10.1186/s12967-023-04771-7)

Additional file 1: Figure. S1. Molecular dynamics simulation of Cucurbitacin E and PI3K. A. The protein is embedded in cubes in the solvent model, the red origin represents the oxygen atom in the water molecule, and the protein is shown in cartoon mode; B, Molecular dynamics simulation results. Protein stability analysis, protein main chain RMSD values. C, protein main chain RMSF values. D, Molecular dynamics simulation results corresponding to the ligand stability analysis: ligand RMSD, ligand RMSF, solvent accessible surface area (SASA), cyclotron radius (RGR). E, MMGBSA binding free energy analysis. F, frequency statistics of interacting amino acids; G, the initial structure (green) and the representative structure (cyan blue) are superimposed.


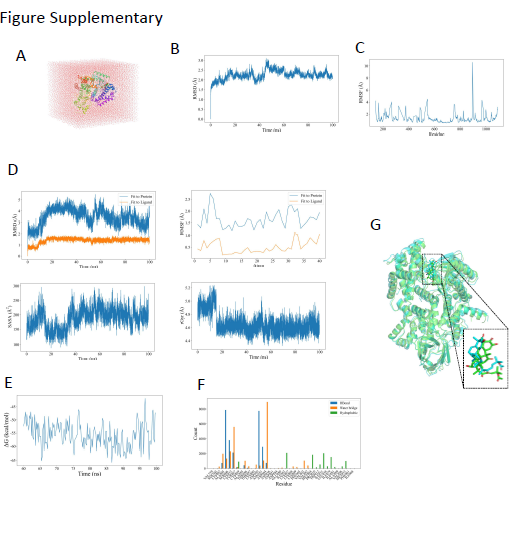

Supplement: Supplementary file 1 — Additional file 1: Figure S1. Molecular dynamics simulation of Cucurbitacin E and PI3K. A The protein is embedded in cubes in the solvent model, the red origin represents the oxygen atom in the water molecule, and the protein is shown in cartoon mode; B Molecular dynamics simulation results. Protein stability analysis, protein main chain RMSD values. C protein main chain RMSF values. D Molecular dynamics simulation results corresponding to the ligand stability analysis: ligand RMSD, ligand RMSF, solvent accessible surface area (SASA), cyclotron radius (RGR). E MMGBSA binding free energy analysis. F frequency statistics of interacting amino acids; G the initial structure (green) and the representative structure (cyan blue) are superimposed. [file 12967_2023_4771_MOESM1_ESM.docx]
